# Comparison of three-dimensional soft tissue changes according to the split pattern after sagittal split osteotomy in patients with skeletal class III malocclusion

**DOI:** 10.1007/s00784-023-05431-2

**Published:** 2023-12-26

**Authors:** Mai Yazaki, Tomoki Aihara, Daigo Okamoto, Shizu Saito, Hikari Suzuki, Shinnosuke Nogami, Kensuke Yamauchi

**Affiliations:** Division of Oral and Maxillofacial Reconstructive Surgery, Department of Oral Medicine and Surgery, Tohoku University Graduate School of Dentistry, 4-1 Seiryo-Machi, Aoba-Ku, Sendai, Miyagi 980-8575 Japan

**Keywords:** Sagittal split osteotomy, 3D soft tissue changes, Split pattern, Long split, Short split, Stability

## Abstract

**Objectives:**

This study aimed to analyse the changes in soft tissue and hard tissue stability associated with the split pattern, i.e. long split (LS) or short split (SS), after sagittal split osteotomy.

**Materials and methods:**

Patients who underwent sagittal split ramus osteotomy were classified into LS or SS groups according to postoperative computed tomography images. They were examined via lateral cephalography and three-dimensional (3D) optical scanning before surgery (T0) and 1 (T1), 3 (T2), and 12 (T3) months after surgery. Six standard angles (SNA, SNB, ANB, FMA, FMIA, and IMPA) were used as measures of hard tissue change. The two sets of 3D data were superimposed, and the volumetric differences were calculated as the soft tissue change. The areas evaluated were delimited by 10 × 20-mm rectangles in the frontal aspect and a 25 × 25-mm square in the lateral aspect.

**Results:**

A total of 42 sides (26 patients) were analysed, including 20 (16 patients) in the SS group and 22 (16 patients) in the LS group. We found no significant differences in cephalographic angle or soft tissue changes in the frontal aspect between the SS and LS groups. We found significant differences in the subauricular region from T0–T1 (*p* = 0.02), T0–T2 (*p* = 0.03), and T0–T3 (*p* = 0.037) in terms of soft tissue changes in the lateral aspect. The volume increase associated with posterior mandibular movement was greater in the LS group.

**Conclusions:**

We found that LS patients with mandibular prognathism exhibited increased subauricular volumes following mandibular setback.

**Clinical relevance:**

It is essential to predict the postoperative facial profile before surgery. The split pattern after sagittal split osteotomy affects the postoperative profile of patients with mandibular prognathism.

## Introduction

The objective of orthognathic surgery is to alter the facial balance to achieve aesthetic results in patients who have severe disharmony of the jaw. Soft tissue structure is an important element of facial aesthetics, which can influence the psychological well-being and quality of life of patients. Thus, oral and maxillofacial surgeons and orthodontists must consider soft tissue contours as well as skeletal relationships and functional occlusion during diagnosis and treatment planning [1]. Postoperative soft tissue morphology is affected by hard tissue movement during orthognathic surgery, and these changes are of great interest to both surgeons and patients [2]. Two-dimensional (2D) cephalograms have previously been used to predict postoperative morphology and, more recently, three-dimensional (3D) images have been employed. Moreover, 3D computed tomography (CT) has been used to simultaneously analyse and measure 3D hard and soft tissue [3]. However, the evaluation of changes in tissue structure at specific times after surgery can be limited by problems associated with tissue irradiation. Other systems, such as 3D lasers and optical surface scans, have been used to assess changes in facial soft tissue [4]. However, these methods also have some limitations, such as the inability to simultaneously view the soft tissue and underlying hard tissue, the requirement for a specific posture during imaging, and the lack of a reliable prediction method [5, 6].

Many studies have reported changes in the hard and soft tissues of patients with skeletal class III malocclusion after orthognathic surgery [3, 7, 8]. However, few studies have conducted 3D evaluations of soft tissue changes after orthognathic surgery in patients with mandibular prognathism, especially in terms of the various effects of isolated mandibular setback. Furthermore, no reports have examined the effects of different surgical techniques on the postoperative facial profile.

**Fig. 1:**
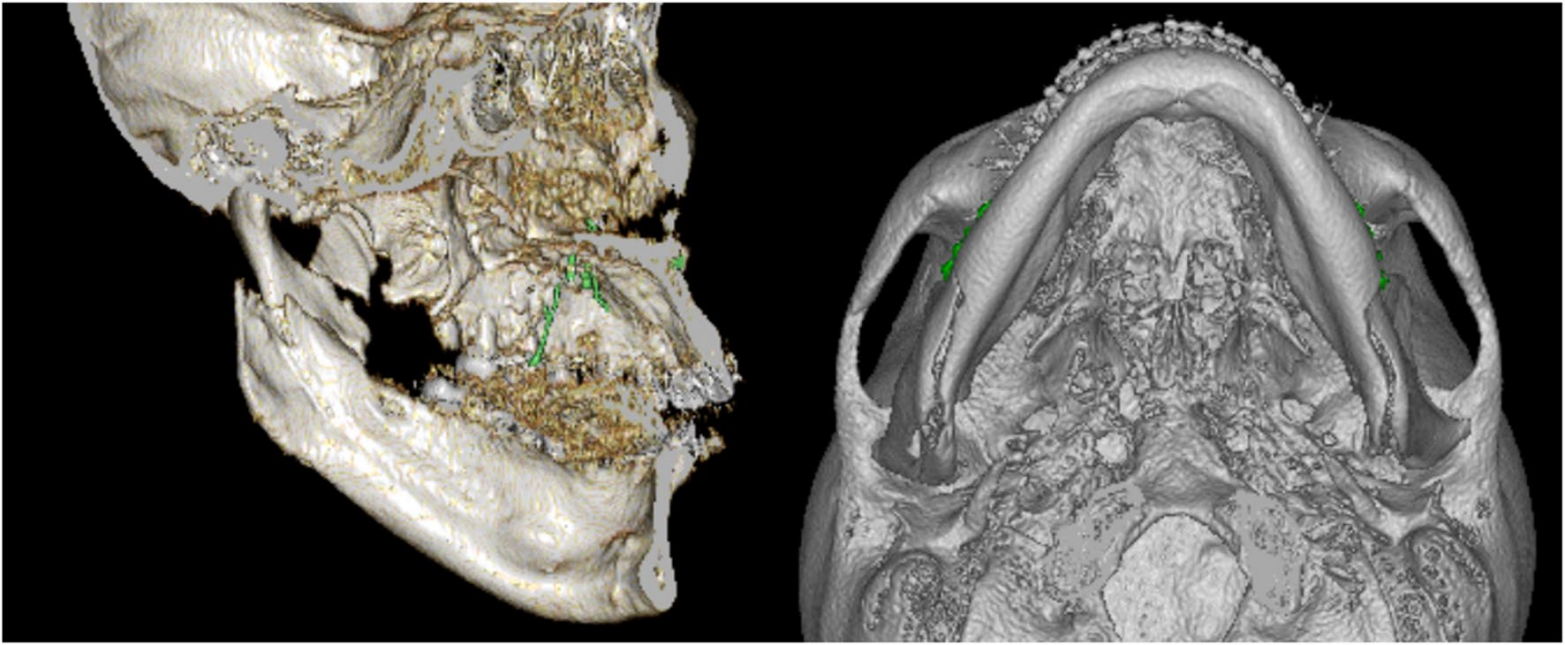
CT images showing LS. A horizontal fracture line extending to the posterior border of the ramus can be seen

**Fig. 2:**
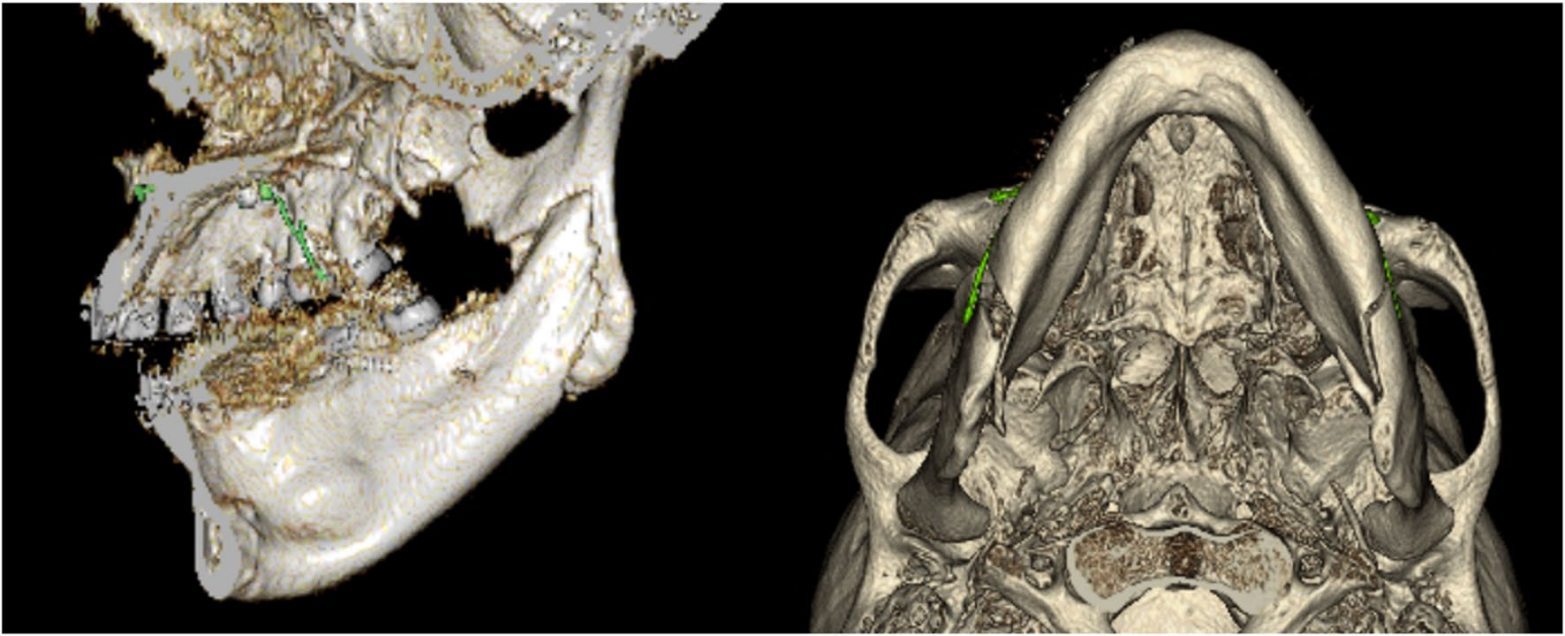
CT images showing SS. A vertical fracture line extending from the lingula to the inferior border of the mandible can be seen

Sagittal split ramus osteotomy (SSRO) can be divided into two types according to the sagittal split pattern: long split (LS; e.g. the Obwegeser method; Fig. 1; [9]) and short split (SS; e.g. the Epker and Wolford methods; Fig. 2 [10, 11]). If an SS is used, the most posterior division is slightly posterior to the mandibular foramen, whereas in an LS, the division extends to the posterior margin of the mandibular ramus. Therefore, the extent of bone overhang from the posterior margin of the mandibular ramus is greater for LS than SS surgery when the mandible is moved posteriorly. However, few reports have addressed the soft tissue changes associated with the LS and SS.

Therefore, the purposes of this study were as follows: (1) to evaluate the stability of hard tissue changes between the LS and SS via conventional 2D cephalograms, and (2) to compare the soft tissue changes between the preoperative assessment and postoperative 12 months using 3D optical scanning to analyse the effect of the mandibular split pattern in patients with mandibular prognathism.

## Materials and methods

### Protocol and patients

This retrospective study was approved by the Ethics Committee of Tohoku University Graduate School of Dentistry. Patients diagnosed with skeletal Class III jaw deformities in the Department of Oral and Maxillofacial Surgery of Tohoku University Hospital, and who underwent SSRO-mediated posterior displacement from January 2017 to December 2019, were included; 20 sides (16 patients) were treated with LSs and 22 (16 patients) with SSs. The exclusion criteria were as follows: bad splits; reoperations; cleft lip or palate; facial scarring; an unclear split position; severe asymmetry (> 4 mm deviation from the midline of the tooth to the midline of the face); and no soft tissue images.

### Surgical procedure

All patients underwent SSRO under general anaesthesia. All surgeries were performed by four skilled oral surgeons. The Dal Pont surgical technique was applied. Using a fissure bar, we made a horizontal medial cut as close to the lingual side as possible. Then, a burr was used to extend the cut anteriorly, medial to the external oblique ridge. Once the second molar tooth was reached, the osteotomy was continued vertically down to the inferior border of the mandible. A thin osteotome was partially mallet-driven into this section, and the split was completed using a Smith superior ramus separator and Smith sagittal split separator.

The mandible was fixed using a six-hole titanium miniplate and screws. For intermaxillary fixation, metal wires or hard elastics were used along with surgical splints for 5 days (both groups). The splints were removed 1–2 weeks after surgery. One evaluator determined SS or LS status using CT images obtained within 1 week after surgery.

### Three-dimensional scanning

Three-dimensional images of facial soft tissues were taken before surgery (T0), and 1 (T1), 3 or 6 (T2), and 12 months (T3) thereafter, using an Artec Eva imaging system (Data Design, Aichi, Japan). Each patient sat with their head in a natural position, with the eyes lightly closed and the lips relaxed. The 3D images were obtained using image reconstruction software (Artec Studio 10; Data Design) and output as STL files.

### Linear and angular analyses

Cephalography was performed by skilled examiners using the UD 150L-40F platform (Shimadzu, Kyoto, Japan) on the same day as CT using the protocol of Tohoku University Hospital (80 kV, 200 mA, irradiation time of 0.1 s, dose of 51.97 mGy/m^2^, focal length of 20 cm, distance from the subject of 200 cm). Acetate paper was used for tracing, and the points used for cephalometric analysis were noted. All tracings were performed twice each by two examiners.

**Fig. 3:**
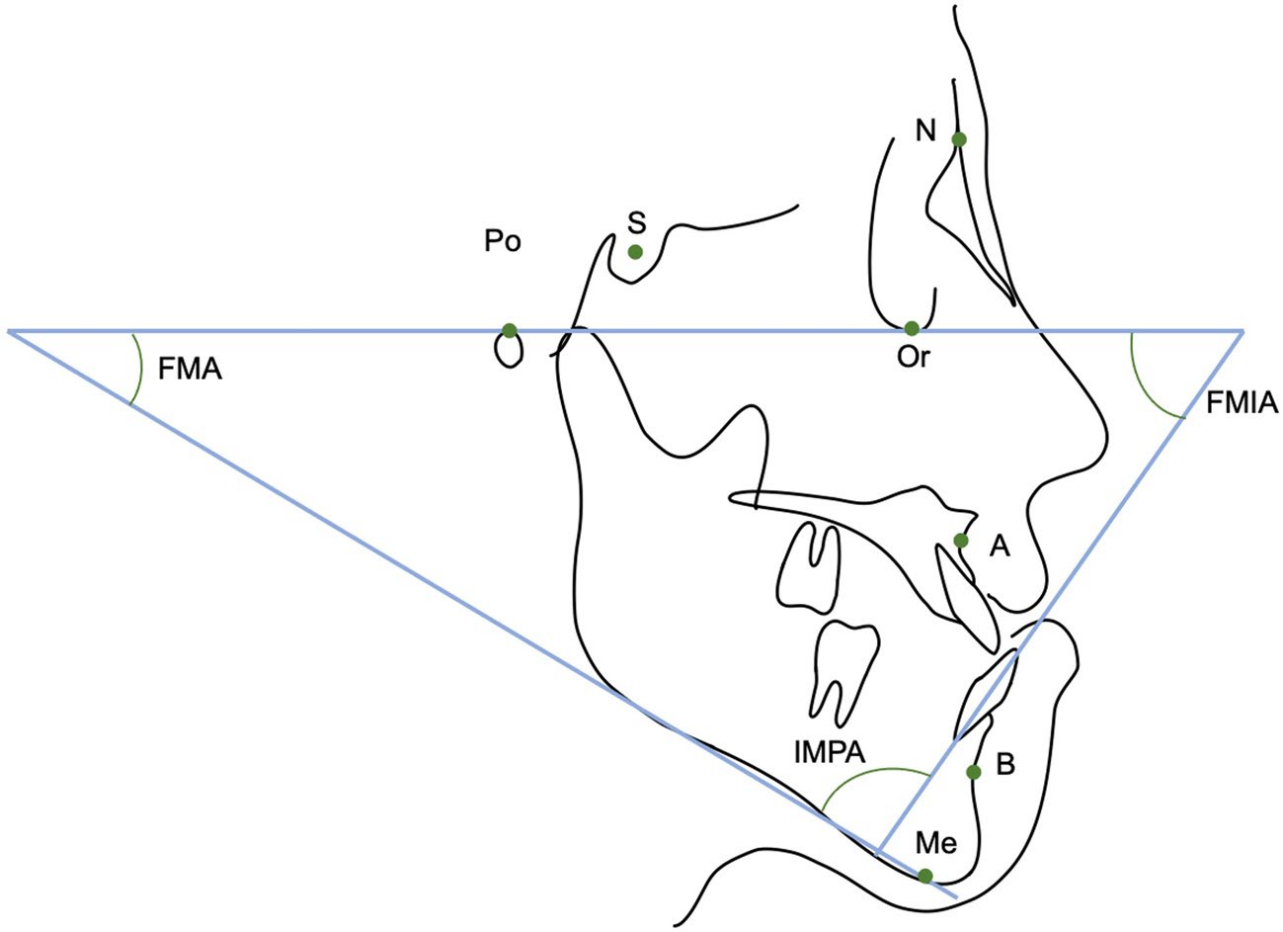
Anatomical landmarks and reference planes used in this study. Sella (S), nasion (N), orbitale (Or), porion (Po), point A (A), point B (B), and menton (Me). IMPA: angle subtended by the mandibular plane and the long axis of the most anterior mandibular incisor. FMA: angle subtended by the Frankfort horizontal plane and the mandibular plane. FMIA: angle subtended by the Frankfort horizontal plane and the long axis of the most anterior mandibular incisor

**Fig. 4:**
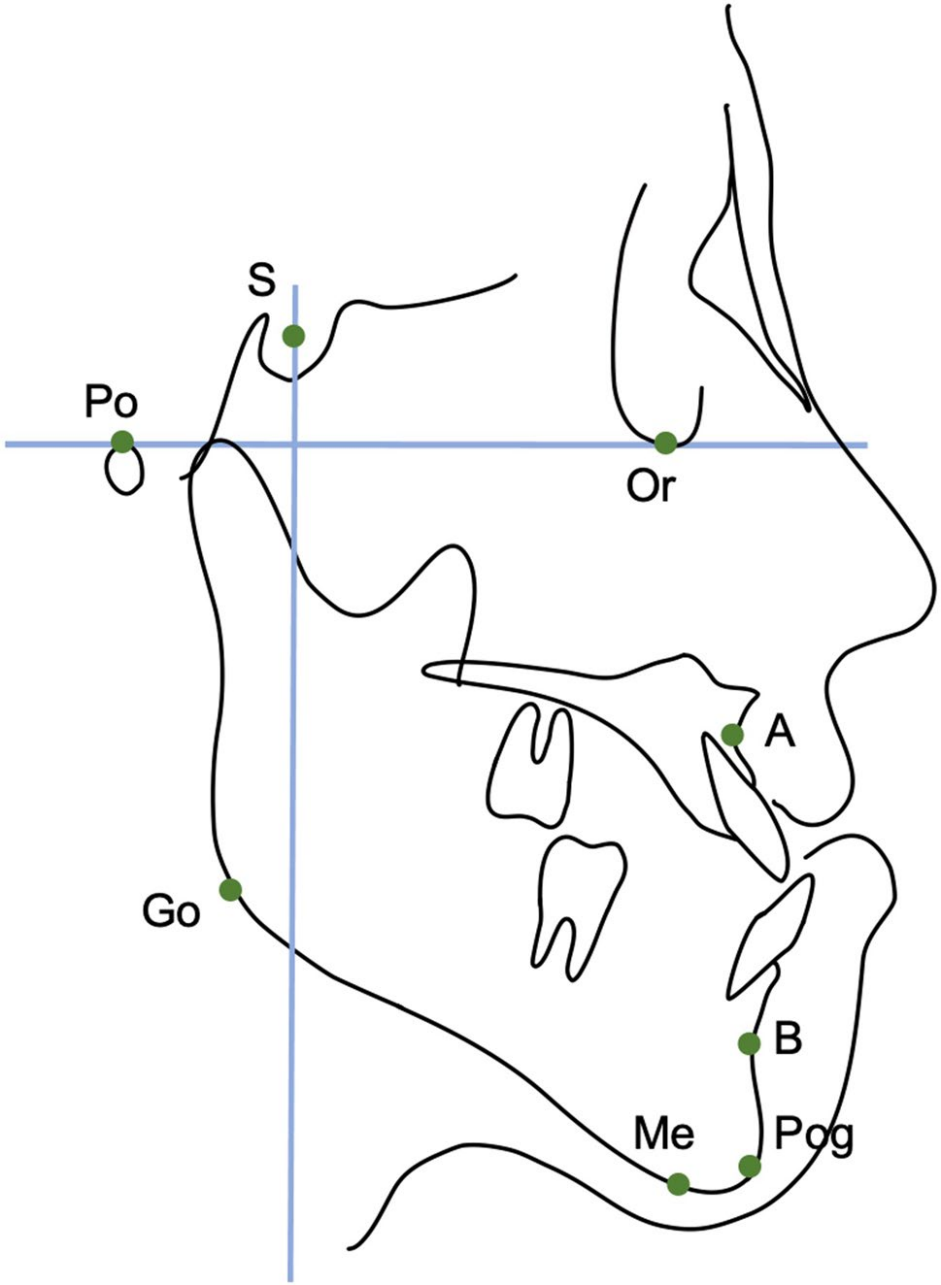
Coordinate axes used in this study. The FH plane served as the x-axis and the perpendicular line through the sella was the y-axis. Changes in the coordinates of A, B, Pog (pogonion), Me, and Go (gonion)

As shown in Fig. 3, we analysed changes in the SNA, SNB, ANB, FMA, FMIA, and IMPA. In terms of the coordinate axis, the FH plane served as the x-axis and the perpendicular line through the sella was the y-axis; changes in the coordinates of A, B, Pog, Me, and Go are shown in Fig. 4.

### Volumetric analysis of 3D data

**Fig. 5:**
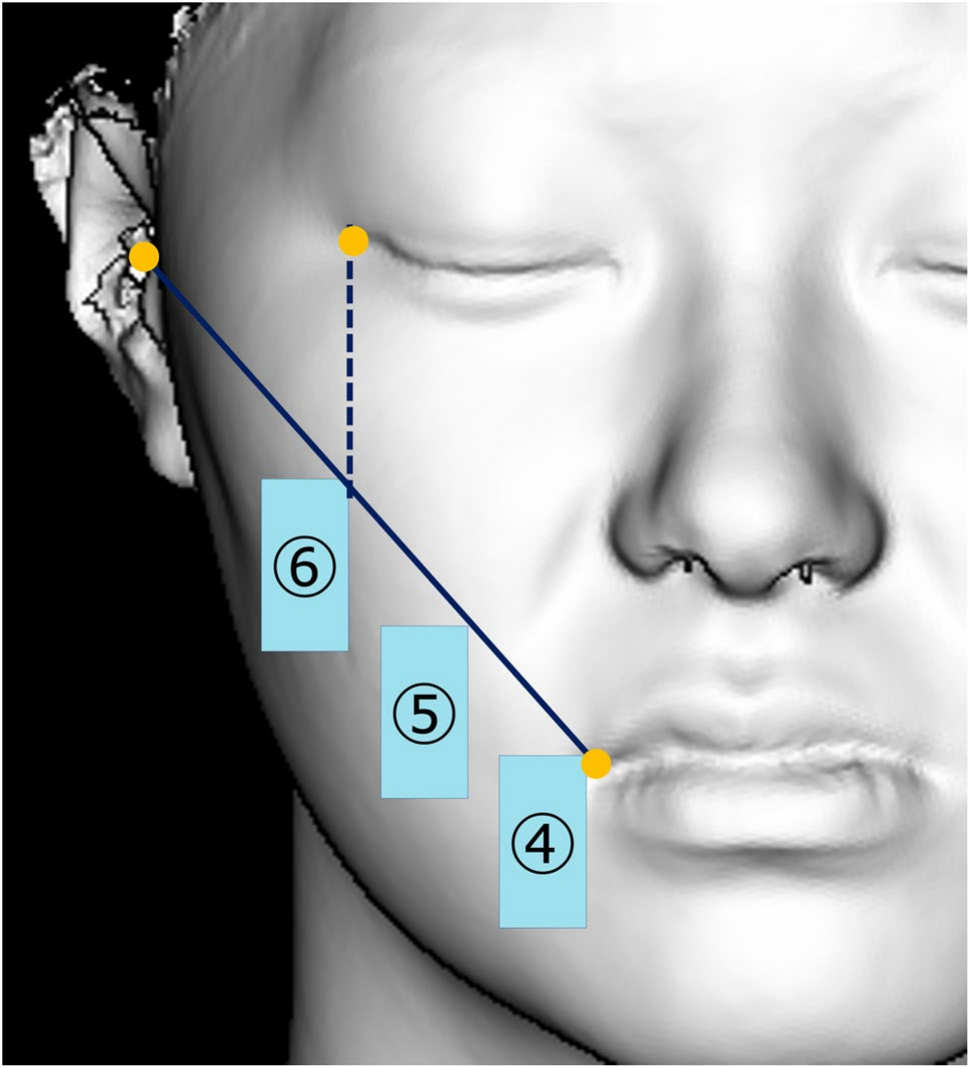
Frontal aspect used for evaluation. The reference line was a straight line connecting the angle of the mouth (cheilion) and the soft tissue porion. Region 4 was defined as the anterior and superior margins of the angle of the mouth (cheilion), and region 6 was defined as the anterior and superior margins of the intersection of the reference line and a perpendicular line drawn from the exocanthion. Region 5 was defined as intermediate between regions 4 and 6

**Fig. 6:**
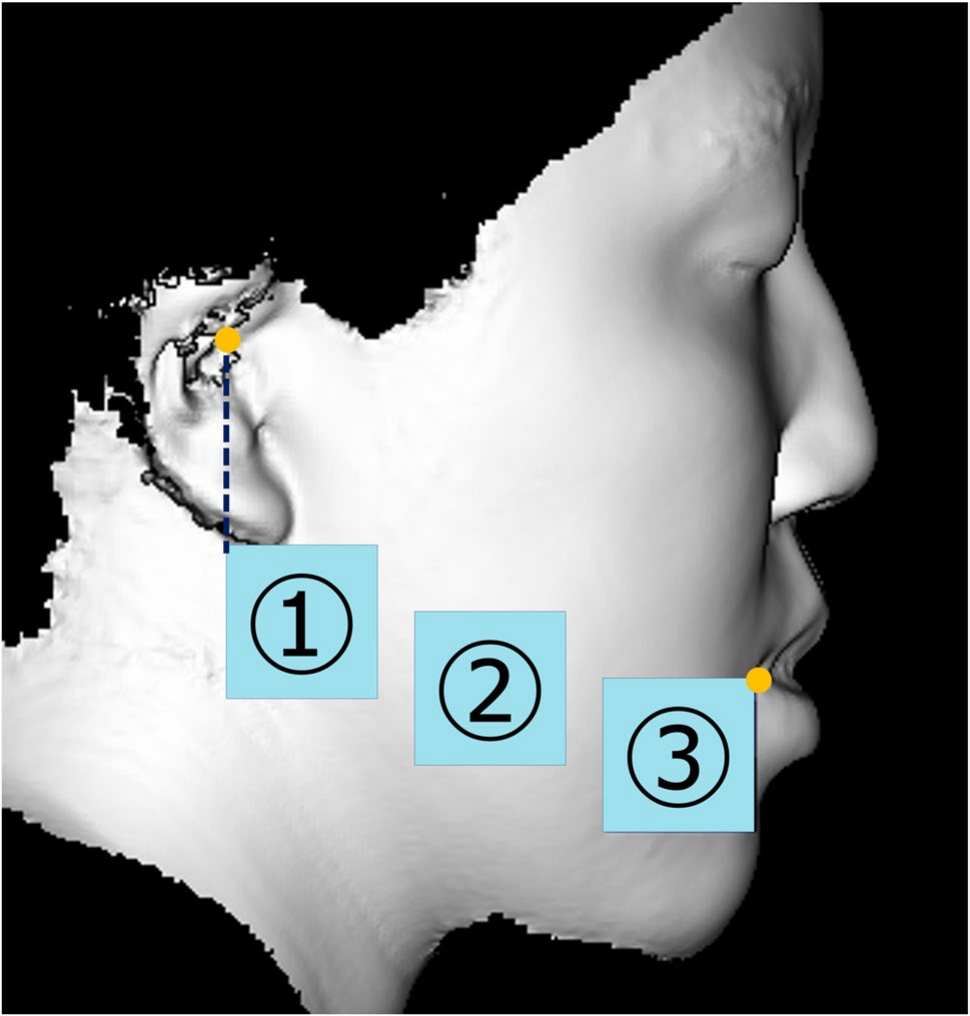
Lateral aspect used for evaluation. Region 1 was defined as the lower part of the auricle (subaurale), with the lower part of the auricle serving as the upper margin and the posterior margin set as a straight line connecting the soft tissue porion. Region 3 was defined as the angle of the mouth (cheilion; the upper and anterior edges), and the area between regions 1 and 3 was considered as region 2 (the cheek area)

STL data were analysed using HBM-Rugle software (Medicoengineering, Kyoto, Japan). For each STL dataset, a vertical plane through the nose and forehead was constructed by reference to the Frankfort horizontal plane. Then, two datasets (from different evaluations) were superimposed by reference to the T-zone area (from the forehead to the root of the nose) [4, 12] using the iterative closest point algorithm. The software calculated volumetric changes in the defined regions by summing the areas showing soft tissue differences in each slice. The areas evaluated were delimited by 10 × 20-mm (vertical × horizontal) rectangles in the frontal aspect (Fig. 5) and a 25 × 25-mm square in the lateral aspect (Fig. 6). We focused on the lower facial region.

### Statistics

Statistical analyses were performed with JMP for Mac software (ver. 16; SAS Institute, Cary, NC, USA). The data are presented as mean ± standard deviation; we compared the groups using the unpaired Student’s *t*-test. *P*-values < 0.05 were considered significant.

## Results

**Fig. 7:**
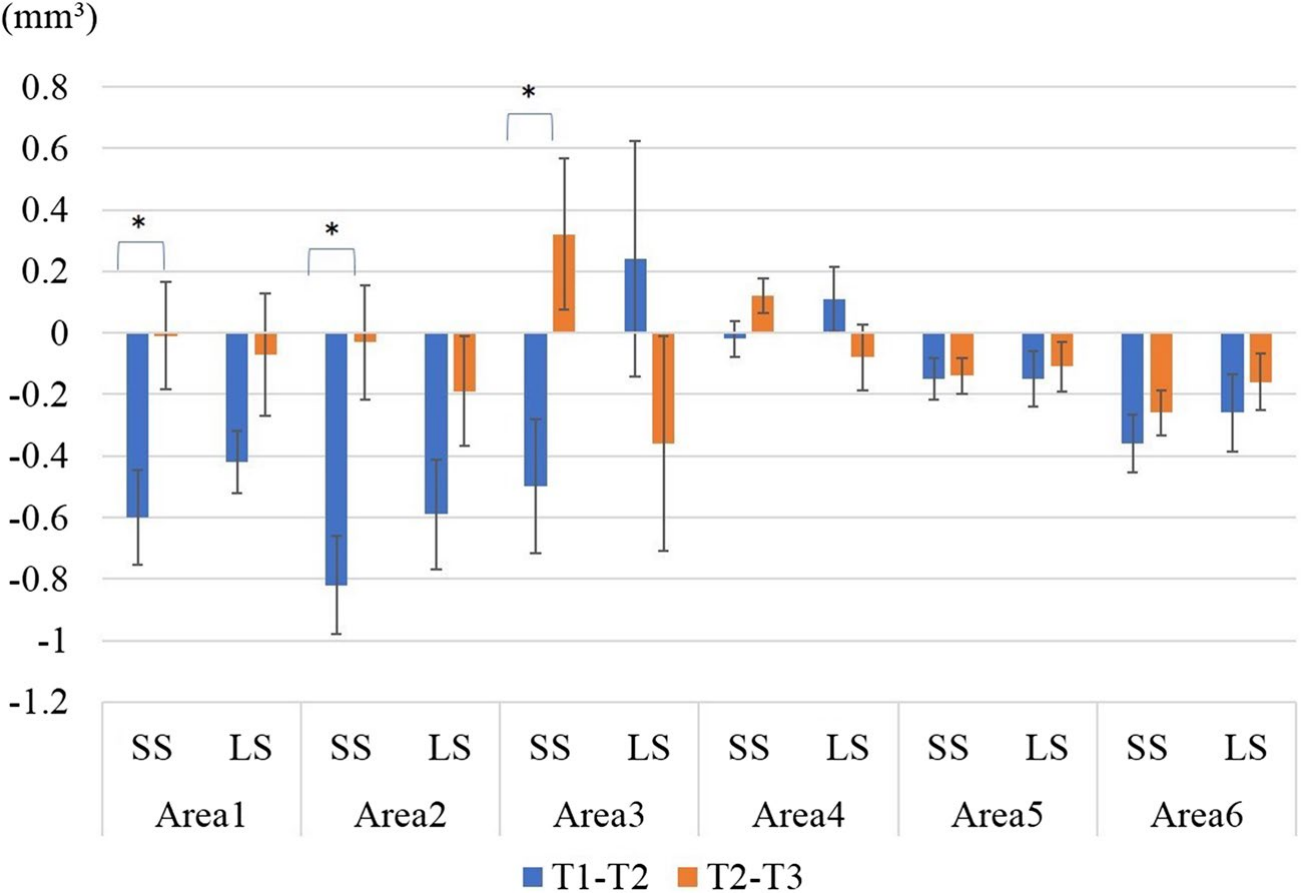
Comparison of T1–T2 and T2–T3. LS; long split, SS; short split, T1; 1 month after surgery, T2; 3 months after surgery, T3; 12 months after surgery, ※; *p*-values < 0.05 were considered indicative of statistical significance

**Fig. 8:**
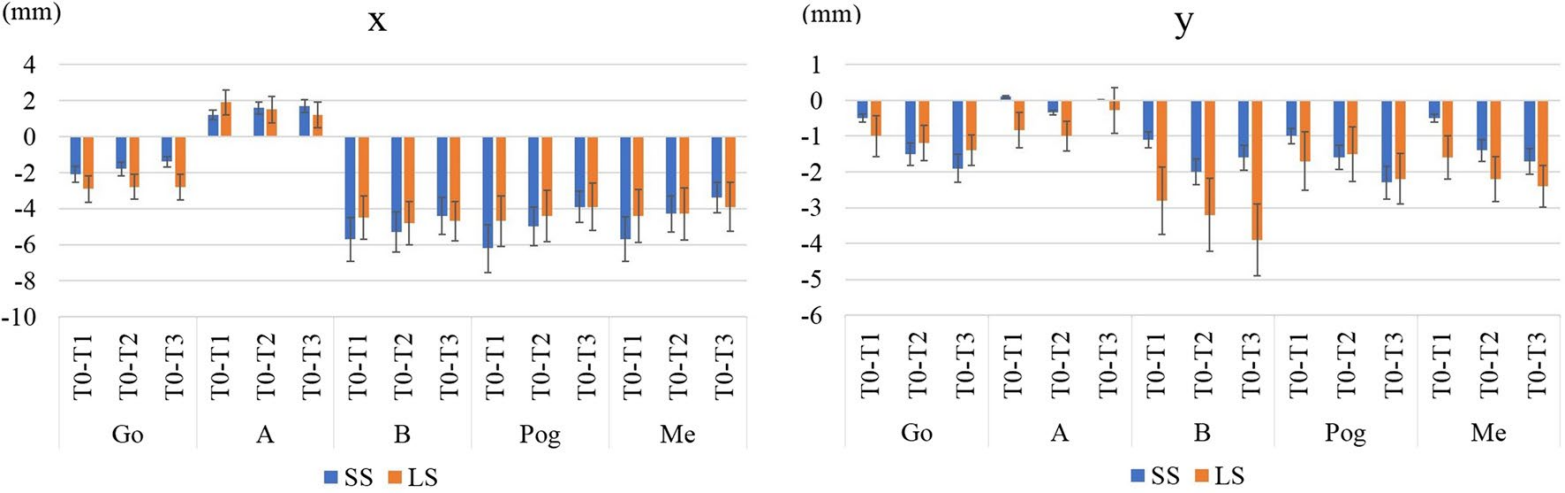
Linear measurements (mm). LS; long split, SS; short split, T0; before surgery, T1; 1 month after surgery, T2; 3 months after surgery, T3; 12 months after surgery. The FH plane served as the x-axis and the perpendicular line through the sella was the y-axis

**Fig. 9:**
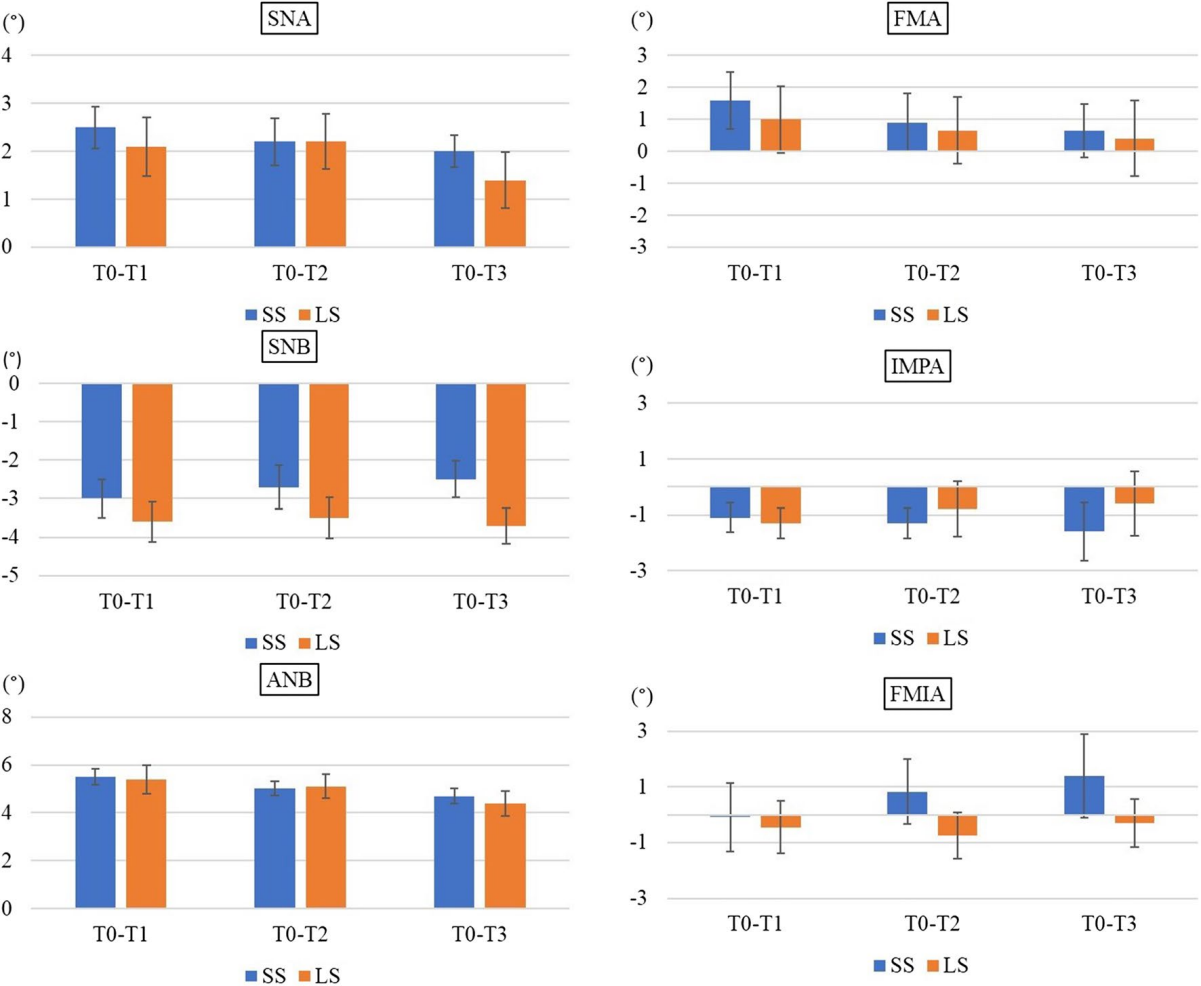
Angular measurements (°).LS; long split, SS; short split, T0; before surgery, T1; 1 month after surgery, T2; 3 months after surgery, T3; 12 months after surgery

A total of 42 sides (26 patients) were analysed, including 20 (16 patients) in the SS group and 22 (16 patients) in the LS group. The mean age was 24.5 ± 6.0 years in the SS group and 21.85 ± 5.0 years in the LS group. Written informed consent was obtained from all patients before surgery (Table 1). There was no significant group difference in age, body mass index (BMI), or the setback volume. Table 2 lists the soft tissue changes apparent in the lateral aspect. In Area 1, significant differences were observed from T0–T1 (*p* = 0.02), T0–T2 (*p* = 0.03), and T0–T3 (*p* = 0.037). The volume increase associated with posterior mandibular movement was clearly greater in the LS group. At T3, the volume of the SS group was the same as that preoperatively, while that of the LS group was higher than the preoperative volume. There was no significant group difference in the volume change in Area 2 or 3. Table 3 lists the soft tissue volume changes in the frontal aspect. Comparison between T1–T2 and T2–T3 revealed significant differences between the two groups in areas 1–3 (Fig. 7). Figures 8 and 9 show the movements of measurement points and changes in cephalographic angles; there were no significant difference between the SS and LS groups.

**Table 1:** Patient’s data (Gender, Age, BMI, Setback)

		SS(n = 22)	LS(n = 20)
Sex		Female; 9, Male; 7	Female; 8, Male; 8
	side	Female; 13, Male; 9	Female; 9, Male; 11
Age(y)		24.5 ± 6.0	21.9 ± 5.0
BMI(Kg/m^2^)		20.7 ± 2.7	21.2 ± 2.6
setback(mm)		7.3 ± 1.8	7.4 ± 2.9

**Table 2:** Amount of change in lateral aspect

Area	T0-T1			T1-T2			T2-T3		
	SS	LS	*p* value	SS	LS	*p* value	SS	LS	*p* value
1	0.64	1.27	0.02*	-0.60	-0.42	0.34	-0.01	-0.07	0.81
2	0.27	0.79	0.66?	-0.82	-0.59	0.35	-0.03	-0.19	0.53
3	-2.19	-2.21	0.86?	-0.50	0.24	0.11	0.32	-0.36	0.12
Area	T0-T2			T1-T3			T0-T3		
	SS	LS	*p* value	SS	LS	*p* value	SS	LS	*p* value
1	0.05	0.85	0.03*	-0.61	-0.49	0.34	0.04	0.78	0.04*
2	-0.19	0.19	0.38?	-0.84	-0.78	0.86	-0.22	0.01	0.66?
3	-2.55	-1.98	0.51?	-0.18	-0.12	0.91	-2.37	-2.33	0.91?

**Table 3:** Amount of change in frontal aspect

Area	T0-T1			T1-T2			T2-T3		
	SS	LS	p value	SS	LS	p value	SS	LS	p value
4	-0.99	-0.72	0.53	-0.02	0.11	0.28	0.12	-0.08	0.12
5	0.04	0.13	0.57	-0.15	-0.15	0.96	-0.14	-0.11	0.83
6	0.40	0.46	0.84	-0.36	-0.26	0.52	-0.26	-0.16	0.39
Area	T0-T2			T1-T3			T0-T3		
	SS	LS	p value	SS	LS	p value	SS	LS	p value
4	-1.01	-0.61	0.34	0.10	0.03	0.67	-0.89	-0.69	0.62
5	-0.11	-0.02	0.57	-0.29	-0.26	0.85	-0.25	-0.13	0.47
6	0.04	0.17	0.52	-0.62	-0.42	0.30	-0.22	0.02	0.25

## Discussion

Orthognathic surgery is used to improve maxillofacial morphology and achieve ideal occlusion; it is very important to predict the postoperative facial profile before surgery. Although 2D soft tissue predictions based on lateral cephalograms have been used in the past, these pertain only to the lateral, i.e. not to the frontal, aspect; moreover, 3D movements of the face are not considered. Over the last few decades, image analysis has made great progress. Hard tissue predictions based on 3D-CT images have greatly improved; however, it remains difficult to accurately predict soft tissue profiles because these tissues are influenced by hard tissue movements, age, body shape, and posture at the time of imaging. Although 3D-CT is often used to simulate the postoperative profile, this is inaccurate when imaging is performed with the patient in the supine position, and cannot be performed frequently because of concerns about radiation exposure.

The optical 3D scanner used in this study was a handheld device that acquires 3D morphologies by shining light on objects of interest, enabling data acquisition in any body position. As there is no radiation, the scanner can be frequently used in the standing and sitting positions to reveal soft tissue changes that occur over time. Studies have compared this technique with manual facial measurements [13], measurements made using 3D-CT images [14], and contact versus non-contact 3D shape measurements [15]. The error in these previous studies was typically within 1 mm. The average error when using our device was acceptable, at 0.02 mm.

We found that the soft tissue volumes increased somewhat from T0–T1 in the lateral aspect and subauricular region, but these differences were significant (*p* < 0.02). The changes may have been caused by swelling occurring immediately after surgery, and by posterior migration in the LS group. The volumes decreased from T1–T2, and from T2–T3, suggesting that swelling improved over time. Bone remodelling proceeded at the edges of bone fragments. We found no obvious difference between the groups, suggesting that the postoperative volume reductions proceeded at the same pace. Significant differences were also observed from T0–T2 (*p* = 0.03) and T0–T3 (*p* = 0.037), suggesting that the initial volume increase was attributable to posterior movement of the distal segment at the posterior border of the mandibular ramus after setback, although the LS group showed a decrease in volume over time. In the SS group, the final volume was the same as that before surgery; setback was associated with almost no volume change.

No significant difference between the SS and LS groups was evident in any area of the frontal region. It is possible that the increased volume of the subauricular region did not significantly affect the frontal aspect.

When the data were compared between T1–T2 and T2–T3, volume loss in the SS group was found to be significantly greater during T1–T2 for all regions (*p* = 0.016 for Area 1, *p* = 0.002 for Area 2, and *p* = 0.017 for Area 3). Areas 1 and 2 in the SS group exhibited very little change in volume after T2; the changes occurring during T2–T3 were very small. Although significant differences were observed in all areas in the LS group, Area 1 showed only a small volume change after T2; the changes during T2–T3 did not differ significantly between the SS and LS groups. Hasegawa et al. found that morphological changes of the posterior margin of the mandibular ramus were completed earlier than changes in the anterior margin, i.e. within 6 months postoperatively [16], consistent with our findings. The soft tissue in Area 3 continued to change after T2 in both the SS and LS groups, which could be due to individual differences in lip relaxation [17, 18] and changes in tooth axes during postoperative orthodontic correction. Changes in soft tissue morphology in Areas 4 and 5 likely persisted for similar reasons. Hasegawa et al. found that the morphology of the anterior margin of the mandibular ramus continued to change beyond 6 months postoperatively [16]. Area 6 is considered to correspond to that margin, which continued to change in volume after T2.

LS osteotomy has been associated with a significantly higher incidence of bad splits and bleeding events compared with SS osteotomy [19]. However, Aoki et al. found no difference in bone healing status on CT between LS and SS patients at 6 months postoperatively [20]. Regarding postoperative neurosensory disturbance (NSD) of the inferior alveolar nerve, some studies reported a lower incidence after SS compared with LS [20, 21], while others found no difference [22]. No report described a higher risk of NSD after SS [23]. Considering our final results regarding soft tissue and hard tissue stability, we recommend SS for patients with pronounced mandibular setback. Additionally, Susarla et al. suggested that a low medial horizontal osteotomy should be performed to avoid NSD in patients with atypical ramus morphology, such as thin proximal ramus with no appreciable marrow space near the lingual aspect or narrow retromolar ramus width [24].

As we excluded cases with significant asymmetry, atypical ramus morphology or congenital deformities, further studies are needed with more patients and greater variation in age, BMI, and the reasons for surgical intervention. Future studies should also incorporate a longer follow-up period.

## Conclusion

The soft tissue profile after orthognathic surgery is an important determinant of patient satisfaction. We found that LS patients with mandibular prognathism exhibited increased subauricular volumes following mandibular setback. However, this was not seen in the SS group; the pre- and post-operative findings were identical at the 1-year follow-up assessment. Thus, the SS technique may offer a stable soft tissue profile after sagittal split osteotomy with mandibular setback. More accurate predictions of soft and hard tissue changes are needed to improve treatments for dentofacial deformities.

## Data Availability

Not applicable.
